# Impact of pulmonary metastasectomy timing and nodule characteristics on survival outcomes in patients with pediatric sarcoma

**DOI:** 10.55730/1300-0144.6070

**Published:** 2025-07-13

**Authors:** İlteriş TÜRK, Mehmet ÇETİN, Neriman SARI, Selma ÇAKMAKCI, Necati SOLAK, Fatma BABACAN, Nesrin GÜRÇAY, Pınar BIÇAKÇIOĞLU

**Affiliations:** 1 Department of Thoracic Surgery, Atatürk Sanatoryum Training and Research Hospital, University of Health Sciences, Ankara, Turkiye; 2 Department of Thoracic Surgery, Etlik City Hospital, University of Health Sciences, Ankara, Turkiye; 3 Division of Pediatric Hematology-Oncology, Department of Pediatrics, Bilkent City Hospital, University of Health Sciences, Ankara, Turkiye; 4 Department of Thoracic Surgery, Ağrı Training and Research Hospital, Ağrı, Turkiye; 5 Department of Pathology, Atatürk Sanatoryum Training and Research Hospital, University of Health Sciences, Ankara, Turkiye

**Keywords:** Pediatric sarcoma, pulmonary metastasectomy, survival

## Abstract

**Background/aim:**

Pulmonary metastasectomy (PM) is a crucial intervention for patients with metastatic sarcomas, particularly among pediatric populations. The timing of PM and its impact on survival outcomes remain a subject of debate in the literature. This study aims to evaluate the impact of PM on survival outcomes.

**Materials and methods:**

This retrospective study included pediatric patients diagnosed with sarcomas who underwent pulmonary metastasectomy. Demographic and clinical characteristics, including age, sex, primary malignancy type, metastasis presence at diagnosis, chemotherapy response, and PM details (e.g., number and laterality of metastatic nodules, time from metastasis detection to surgery) were analyzed. Survival data were assessed using Kaplan–Meier curves and Cox regression analysis, and statistical significance was determined using log-rank tests.

**Results:**

A total of 29 patients (51.7% male, 48.3% female) were included, with 55.2% presenting with metastasis at diagnosis. Overall, 47 surgical procedures were performed. The 5-year survival rate following PM was significantly higher for patients who underwent surgery within 30 days of metastasis detection (75.26%) compared to those who had surgery later (35.88%, p = 0.031). In univariate analysis, both 5-year survival after nodule detection and 5-year survival after metastasectomy were better in patients who underwent PM within the first 30 days (p = 0.022 and p = 0.039, respectively). Additionally, the number of metastatic nodules (< 10 versus ≥ 10) and the number of nodules per operation (< 3 versus ≥ 3) were significant factors influencing survival. Patients with fewer metastatic nodules exhibited better 5-year survival rates. The survival outcomes were comparable across different sarcoma subtypes, including osteosarcoma, Ewing sarcoma, and synovial sarcoma.

**Conclusion:**

Our study suggests that early pulmonary metastasectomy, performed within 30 days of metastasis detection, improves 5-year survival in pediatric sarcoma patients. The number of metastatic nodules and the timing of surgery are critical factors in determining survival outcomes. These findings highlight the importance of timely surgical intervention and careful evaluation of metastasis extent in optimizing patient prognosis.

## 1. Introduction

Pulmonary metastasectomy (PM) has long been employed in the management of lung metastases originating from various primary organ cancers. Over the years, several criteria for surgical candidacy have gained general acceptance. These include control of the primary tumor, absence of extrapulmonary metastases, complete resectability of pulmonary metastases, adequate pulmonary reserve to tolerate surgery, and the absence of a more effective alternative treatment option. Numerous studies have emphasized that PM performed in accordance with these patient selection criteria can significantly improve survival outcomes [1–3].

Malignancies in children represent a specific subgroup within this context. In pediatric patients with solid tumors, metastatic disease and complications of its treatment are the most common causes of mortality. Among children with bone and soft tissue malignancies, the incidence of pulmonary metastases at diagnosis or during follow-up is particularly high [4,5]. Since Richardson’s 1961 publication of outcomes following PM in 35 pediatric patients, the literature has consistently advocated that staged bilateral resections should not be avoided, that the number of metastases and the disease-free interval should not be considered absolute contraindications for PM, and that the primary histology is the most critical determinant of survival [6–8].

This study aims to evaluate the prognostic factors influencing survival following PM in pediatric patients with sarcomas and to assess the impact of timing of PM on clinical outcomes.

## 2. Materials and methods

A retrospective analysis was conducted on data from 29 pediatric patients who underwent pulmonary metastasectomy (PM) for lung metastases of bone and soft tissue sarcomas at our center between 2016 and 2022. Metastasectomies performed in adult patients and procedures targeting nonsarcoma tumor metastases in pediatric patients were excluded from the study. Local ethics committee approval was obtained prior to the study (Approval no: 2024-BÇEK/56), and the study was conducted in accordance with the Declaration of Helsinki. Patient data included age, sex, histologic subtype of the primary tumor, presence of pulmonary metastases at the time of initial diagnosis, time interval between detection of the pulmonary nodule and PM (categorized as surgery within the first month versus later), laterality of PM (unilateral or bilateral), number of PM procedures, total number of resected metastatic nodules per procedure (categorized as < 3, 3–10, or > 10), receipt of radiotherapy (RT) to the lungs, presence of complications, length of hospital stay, postmetastasectomy survival time, survival time from nodule detection, and 5-year survival rates. The impact of each variable on survival time and 5-year survival rate, both after PM and from the time of nodule detection, was evaluated.

### 2.1. Surgical technique and management

All patients included in the study were evaluated for pulmonary metastases via thoracic computed tomography (CT) during follow-up in the pediatric oncology clinic where they were being treated for their primary tumor. Positron emission tomography/computed tomography (PET/CT) was performed at diagnosis and at relapse to evaluate extrapulmonary metastases. Once the decision for PM was made by the oncology team, patients were referred to the thoracic surgery department, and the preoperative preparation process was initiated. The surgical team assessed whether the patients’ primary tumors were under control, whether all pulmonary nodules could be resected with PM, and whether the patients had sufficient respiratory capacity. Preoperative anesthesia evaluations were initiated for patients deemed suitable. All patients met the widely accepted criteria for PM eligibility.

All surgeries were performed under general anesthesia using double-lumen endotracheal intubation, with patients positioned in the lateral decubitus position, and a muscle-sparing thoracotomy approach was used. When necessary, staged bilateral thoracotomies or repeated thoracotomies on the same side were performed due to newly developed nodules during follow-up. In patients with numerous small nodules, excision was performed using the precision cautery excision technique as described by Perelman [9]. For fewer and larger nodules, wedge resections were performed. All resections were nonanatomical, and no patient required anatomical resection. At the end of each operation, a single chest drain was routinely placed. In the absence of complications, patients were monitored for 1 day in the intensive care unit and then transferred to the general ward. They were discharged following control chest radiography on the day the drain was removed.

### 2.2. Chemotherapy protocols

For osteosarcoma, first-line chemotherapy consisted of a combination of high-dose methotrexate, doxorubicin, and cisplatin (MAP regimen) [10]. In relapsed or refractory cases, second-line therapies included high-dose ifosfamide, oxaliplatin, irinotecan, and the multikinase inhibitor sorafenib [11].

Patients with Ewing sarcoma received alternating cycles of vincristine, doxorubicin, and cyclophosphamide (VDC) with ifosfamide and etoposide (IE) as standard first-line treatment [12]. In cases of relapse or refractory disease, salvage regimens containing irinotecan, temozolomide, carboplatin, and tyrosine kinase inhibitors were used [13].

In rhabdomyosarcoma, low- and intermediate-risk patients were treated with vincristine, dactinomycin, and cyclophosphamide (VAC). For high-risk patients, this regimen was intensified with vincristine, doxorubicin, and cyclophosphamide (VDC), alternating with ifosfamide and etoposide (IE), and supplemented with irinotecan [14]. Relapsed or refractory cases were managed with carboplatin-based chemotherapy and tyrosine kinase inhibitors [15]. For patients with synovial sarcoma and alveolar soft part sarcoma, first-line chemotherapy included ifosfamide and doxorubicin, while tyrosine kinase inhibitors were used in relapsed patients.

### 2.3. Statistical analysis

All statistical analyses were performed using SPSS software, version 24.0 (IBM Corp., Armonk, NY, USA). Descriptive statistics were presented as count (n), percentage (%), mean ± standard deviation (SD) for age, and median ± standard error (SE) for survival durations. Overall survival from the time of surgery, from the time of nodule detection, and from initial diagnosis was evaluated using the Kaplan–Meier method and compared using the log-rank test. Variables analyzed included age, sex, presence of metastasis at diagnosis, primary malignancy type, response to chemotherapy, primary relapse, pulmonary relapse, interval between metastasis detection and surgery, surgical approach, number of surgeries, total number of nodules, number of nodules per surgery, receipt of pulmonary radiotherapy, and presence of postoperative complications. The 2-year and 5-year survival rates after surgery and after nodule detection were also calculated using the Kaplan–Meier method and compared across groups. Survival time after PM and from nodule detection was compared with other parameters using Cox regression analysis. A p-value less than 0.05 was considered statistically significant.

## 3. Results

Of the 29 patients, 15 were female and 14 were male, with a mean age of 13.59 ± 3.96 years (range: 3–18 years). The most common primary malignancy leading to pulmonary metastasis was osteosarcoma, accounting for 41.4% of cases. The primary tumors were most frequently located in the lower extremities, predominantly in the femur. The mean interval from initial diagnosis of the primary tumor to the development of pulmonary metastases was 13.3 months (range: 0–64 months). A total of 47 PM procedures (range: 1–4 per patient) were performed. Among the 864 pulmonary nodules excised during these surgeries, 782 (90.5%) were histopathologically confirmed as metastatic. The median number of nodules resected per surgery was six (range: 1–72). The median time from detection of pulmonary nodules to PM was 90 days (range: 2–870 days). Postoperative complications occurred in four (8.5%) of the 47 operations. There was no surgical mortality. The median length of hospital stay following PM was 5 days (range: 3–9 days). Demographic and general perioperative data are summarized in Table 1.

Patients aged 12 years or younger, those who underwent PM within the first 30 days following nodule detection, those with fewer than 10 total metastatic nodules, and those with fewer than three nodules excised per procedure had significantly longer survival after PM compared to other groups. No statistically significant differences in survival were observed among other parameters (Table 2).

**Figure 1:**
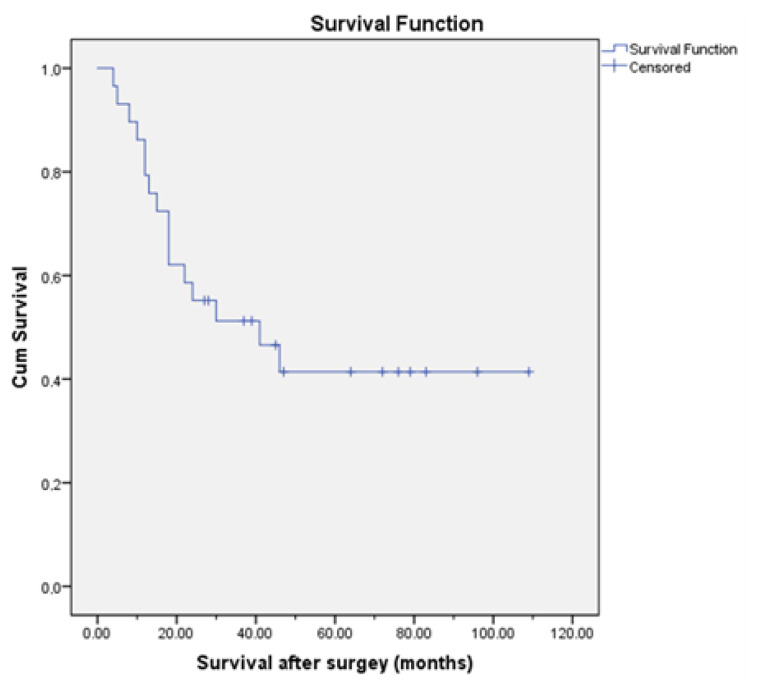
Kaplan–Meier curves for 5-year survival rates (40.7% ± 10%) from the time of nodule detection.

**Figure 2:**
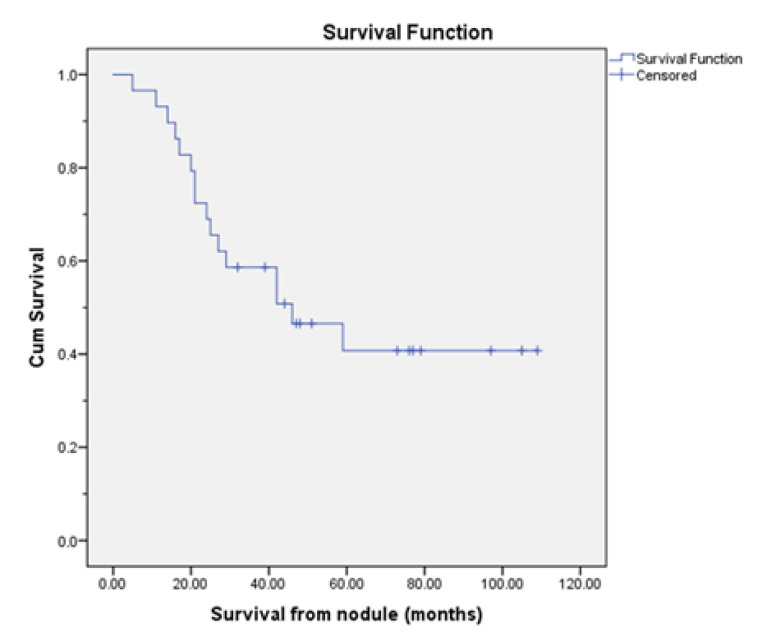
Kaplan–Meier curves for 5-year survival rates (41.4% ± 9.8%) after pulmonary metastasectomy (PM).

With a median follow-up duration of 42 months, 11 patients were alive, while 18 patients (62.1%) had died. At the time of analysis, the mean post-PM survival duration was 51.97 ± 7.98 months, while the mean survival from the time of nodule detection was 57.52 ± 7.50 months. The 5-year survival rate from the time of nodule detection was 40.7% ± 10%, and the 5-year survival rate following PM was 41.4% ± 9.8%. Kaplan–Meier survival curves for 5-year survival rates are presented in Figure 1 and Figure 2.

Analysis of prognostic factors revealed that patients with fewer than three nodules resected per PM procedure and those who underwent PM within 30 days of nodule detection had significantly higher 5-year survival rates both after PM and from the time of nodule detection (p = 0.006 and p = 0.039, respectively) (Table 3). In multivariate Cox regression analysis, post-PM survival was found to be significantly better in patients whose interval between metastasis and surgery was less than 30 days. However, no statistically significant difference was observed in survival time following nodule detection (p = 0.035 and p = 0.150, respectively) (Table 4).

## 4. Discussion

The optimal timing of pulmonary metastasectomy (PM) has long been debated in the literature, with varying findings reported. Tanaka et al., in a study including both adult and pediatric patients with diverse primary histologic types, suggested that a 3-month surveillance period following the detection of pulmonary nodules might help prevent early relapse and improve prognosis [16]. In contrast, another study focusing solely on pulmonary metastases from colorectal cancers recommended a considerably longer follow-up period of 9 months after nodule detection [17]. Yet another study on colorectal cancer metastases proposed immediate PM for solitary and peripheral lesions, while recommending two consecutive 3-month follow-up intervals before deciding on PM or deferring surgery in other cases [18]. On the other hand, Detterbeck et al., in a publication evaluating imaging requirements before PM, advocated for the earliest possible surgical intervention if the patient’s clinical condition allows, arguing that delaying resection offers no benefit [19]. Waiting for PM after diagnosis may be considered questionable due to the possibility of existing metastases growing and requiring more extensive resections or even seeding additional metastases.

In a comprehensive review, Krüger summarized the main arguments against early surgery in three categories: (1) deferring surgery to exclude rapidly progressive disease during the initial months, (2) allowing for chemotherapy administration before PM, and (3) avoiding reoperations in case of initially undetected small lesions [20]. However, numerous studies have emphasized that repeat surgeries do not adversely affect survival and should not be avoided when necessary [21–23]. An earlier study focusing on pediatric sarcomas also supported the survival benefit of repeat PM in appropriate cases [24]. In a study involving 81 sarcoma patients, adjuvant chemotherapy following R0 resection during PM did not demonstrate additional survival benefit [25]. A study examining the doubling time of pulmonary metastases reported 109 days for colorectal cancers, 42 days for sarcoma metastases, and only 37 days for very young patients regardless of histologic type [26]. In our study, univariate analysis showed that performing PM within the first 30 days after nodule detection significantly improved both 5-year survival from nodule detection and post-PM survival. In multivariate analyses, while the post-PM outcome remained statistically significant, the lack of statistical significance for survival after nodule detection may be attributed to the limited number of patients. Therefore, we advocate for prompt PM in pediatric patients with sarcoma who meet surgical criteria.

The timing of metastasis appearance may also provide prognostic insights. In a study evaluating 88 osteosarcoma patients who underwent PM, patients were grouped based on the timing of metastasis: present at initial diagnosis, emerging during chemotherapy, or appearing during follow-up after completion of treatment. The worst survival outcomes were observed in patients who developed metastases during chemotherapy [27]. Another study with a smaller osteosarcoma cohort suggested that the presence of pulmonary metastases at diagnosis was associated with worse survival [28]. A separate study identified pulmonary metastases progressing under neoadjuvant chemotherapy as an independent prognostic factor for poor survival [25]. In our study, patients were grouped based on whether they had pulmonary metastases at initial diagnosis. While the 5-year survival rate was 45.8% in those with metastases at diagnosis and 36.9% in those without, the difference was not statistically significant. This may be due to the heterogeneity of the second group, as also noted by Ahmed et al. [27].

The number of pulmonary metastases has long been a subject of debate in terms of its impact on survival, and PM is increasingly being performed in patients with multiple metastases. Nevertheless, a high number of nodules is often considered a marker of aggressive tumor biology [29]. In a study of 615 patients undergoing PM for colorectal cancer, those with three or fewer metastatic nodules had significantly better 5-year survival rates [30]. Similarly, a study on sarcoma metastases found that progression under chemotherapy and the presence of more than three metastases were independent risk factors for worse overall survival [31]. Consistent with the literature, we found that the presence of fewer than three metastatic nodules per operation, regardless of the total number of surgeries, was significantly associated with longer survival and higher survival rates in our patient cohort.

Smolle et al., in their study on lung metastases of bone sarcomas, reported that the majority of patients had osteosarcoma, followed by Ewing sarcoma, and found no significant survival differences between histologic types. They reported a 5-year survival rate of 44.8% after PM [32]. Lin et al., in another study, reported a median survival of 35.4 months and a 5-year survival rate of 34.8%. Conversely, they noted better survival after PM in osteosarcoma compared to synovial sarcoma [33]. Another study reporting a 5-year survival rate of 41% demonstrated the survival benefit of PM in relapsed osteosarcoma cases [34]. Osteosarcoma and Ewing sarcoma—the most common and second most common bone tumors in children, respectively [35]—constituted the majority of our cohort. However, we found no statistically significant difference in survival between different histologic subtypes. Our 5-year survival rate of 41.4% after PM is consistent with previously reported data.

A current topic of discussion is whether thoracotomy or video-assisted thoracoscopic surgery (VATS) yields better outcomes in PM. Many recent studies report that VATS does not confer a survival disadvantage compared to thoracotomy. A metaanalysis including 1541 patients from four studies found no survival difference between VATS and thoracotomy, while VATS was associated with shorter hospital stays [36]. Another study based on database analysis noted that although VATS use was associated with shorter hospitalizations, the rate of adoption demonstrated a peak in 2009 [37]. In a survey conducted by the American Pediatric Surgical Association, only 34% of surgeons preferred VATS for patients with three nodules on one side, and this dropped to 21% for five nodules [38]. A study comparing PM via thoracotomy and VATS in 202 osteosarcoma patients found similar survival outcomes but expressed concern over potential selection bias. Additionally, although not statistically significant, the VATS group had a higher number of R1 resections [39]. A study conducted in Türkiye including 226 PM procedures (including adults) found that thoracotomy allowed resection of 1.9 times more malignant nodules than were initially detected on CT scans [40]. There is a common concern that subcentimeter nodules may be missed using VATS due to the lack of bimanual palpation, potentially resulting in incomplete (non-R0) resections. Moreover, the inability of CT to detect some of these small nodules further emphasizes the importance of manual palpation. For all these reasons, we prefer and recommend a muscle-sparing thoracotomy approach for PM in accordance with oncologic principles.

Although our study included only pediatric sarcoma cases, its limitations include its single-center nature, a relatively small sample size, exclusion of patients with pulmonary metastases who did not undergo surgery, limited generalizability due to the inclusion of multiple histological subtypes in a single analysis, and the retrospective design, which may have introduced selection bias. Nevertheless, our findings contrast with some previous literature and demonstrate that, in pediatric sarcoma patients who meet surgical criteria, early PM contributes to improved survival.

In conclusion, early pulmonary metastasectomy, particularly when performed within 30 days of nodule detection, appears to improve survival in pediatric sarcoma patients. A lower number of metastatic nodules per surgery also correlates with better outcomes. These findings support expeditious surgical intervention in eligible cases to maximize long-term survival.

## Figures and Tables

**Table 1:** Demographic and general clinical characteristics of the patients.

Parameters	Number	Percentage (%)

Sex		
Female	15	51.7
Male	14	48.3

Age (years)		
≤12	9	31
>12	20	69

Primary malignancy		
Osteosarcoma	12	41.4
Ewing sarcoma	8	27.6
Synovial sarcoma	4	13.8
Rhabdomyosarcoma	4	13.8
Alveolar soft part sarcoma	1	3.4

Presence of metastasis at initial diagnosis		
Present	16	55.2
Absent	13	44.8

Chemotherapy response		
Complete	23	79.3
Partial	6	20.7

Laterality of PM		
Unilateral	17	58.6
Bilateral	12	41.4

Number of PM procedures		
1	16	55.2
2	9	31
3	3	10.3
4	1	3.4

Pulmonary radiotherapy		
Absent	19	65.5
Present	10	34.5

Postoperative complications (per procedure)		
None	43	91.5
Prolonged air leak	2	4.3
Pleural effusion	1	2.1
Subcutaneous hematoma	1	2.1

**Table 2:** Comparison of various parameters with respect to survival time after pulmonary metastasectomy.

Parameters	Subtype		Mean				p-value

			Estimate	Std. error	95% Confidence interval		
	
		Number (n)			Lower bound	Upper bound	

Sex	Male	14 (48.3%)	45.29	10.80	24.12	66.45	0.433
	Female	15 (51.7%)	58.60	11.63	35.81	81.40	

Age (years)	≤12	9 (31.0%)	84.50	15.00	55.09	113.91	**0.039**
	>12	20 (69.0%)	40.13	7.81	24.82	55.45	

Primary malignancy	Osteosarcoma	12 (42.9%)	37.96	9.74	18.87	57.05	0.517
	Ewing sarcoma	8 (28.6%)	64.25	16.80	3.33	97.17	
	Synovial sarcoma	4 (14.3%)	37.75	14.53	9.27	66.23	
	Rhabdomyosarcoma	4 (14.3%)	59.50	8.02	31.49	87.51	

Presence of metastasis at initial diagnosis	Present	16 (55.2%)	58.46	10.90	37.10	79.82	0.341
	Absent	13 (44.8%)	44.42	11.92	21.05	67.79	

Chemotherapy response	Partial	6 (20.7%)	36.33	12.83	11.18	61.48	0.281
	Complete	23 (79.3%)	58.93	9.83	39.66	78.21	

Interval between metastasis detection and surgery	First 30 days	10 (34.5%)	75.26	11.38	52.96	97.56	**0.031**
	>30 days	19 (65.5%)	35.88	7.36	21.47	50.30	

Laterality of PM	Unilateral	17 (58.6%)	53.31	7.96	37.70	68.92	0.179
	Bilateral	12 (41.4%)	39.14	10.78	18.02	60.26	

Number of PM procedures	1	16 (55.2%)	55.52	8.15	39.55	71.50	0.109
	>1	13 (44.8%)	37.51	10.07	17.78	57.25	

Total number of metastatic nodules	<10	15 (53.6%)	66.52	10.41	46.11	86.93	**0.032**
	≥10	13 (46.4%)	28.46	6.09	16.53	40.40	

Number of nodules per procedure	<3	9 (32.1%)	75.14	12.43	50.78	99.49	0.054
	≥3	19 (67.9%)	40.08	9.74	21.00	59.16	

Number of nodules per patient	<3	9 (32.1%)	75.14	12.43	50.78	99.49	**0.009**
	≥3 and ≤10	9 (32.1%)	56.89	15.04	27.42	86.36	
	>10	10 (35.7%)	19.59	3.84	12.06	27.12	

Pulmonary radiotherapy	Present	10 (34.5%)	45.60	14.10	17.96	73.24	0.286
	Absent	19 (65.5%)	57.77	9.94	38.29	77.25	

Postoperative complications (per procedure)	Present	25 (86.2%)	23.75	6.82	10.38	37.12	0.433
	Absent	4 (13.8%)	54.25	8.65	37.31	71.20	

**Table 3:** Effects of various parameters on the 5-year survival rate after PM and from the time of nodule detection.

Parameters	Subtype	Number (n)	Five-year survival rate after surgery (%)	p-value	Five-year survival rate from the nodule (%)	p-value

Primary malignancy	Osteosarcoma	12 (42.9%)	25.0 ± 12.5	0.505	25.0 ± 12.5	0.454
	Ewing sarcoma	8 (28.6%)	62.5 ± 17.1		60.0 ± 18.2	
	Synovial sarcoma	4 (14.3%)	25.0 ± 21.7		25.0 ± 21.7	
	Rhabdomyosarcoma	4 (14.3%)	75.0 ± 21.7		75.0 ± 21.7	

Presence of metastasis at initial diagnosis	Present	16 (55.2%)	46.3 ± 13.5	0.355	45.8 ± 13.5	0.315
	Absent	13 (44.8%)	34.6 ± 14.4		36.9 ± 13.8	

Chemotherapy response	Partial	6 (20.7%)	16.7 ± 15.2	0.342	16.7 ± 15.2	0.197
	Complete	23 (79.3%)	49.6 ± 11.0		47.3 ± 11.7	

Interval between metastasis detection and surgery	First 30 days	10 (34.5%)	67.5 ± 15.5	0.022	67.5 ± 15.5	0.039
	>30 days	19 (65.5%)	29.5 ± 11.0		24.1 ± 12.3	

Laterality of PM	Unilateral	17 (58.6%)	57.5 ± 12.3	0.119	52.9 ± 13.9	0.109
	Bilateral	12 (41.4%)	22.2 ± 12.8		25.0 ± 12.5	

Number of PM procedures	1	16 (55.2%)	61.1 ± 12.6	0.068	56.3 ± 14.4	0.051
	>1	13 (44.8%)	20.5 ± 12.0		23.1 ± 11.7	

Total number of metastatic nodules	<10	15 (53.6%)	57.0 ± 13.5	0.032	59.3 ± 12.9	0.059
	≥10	13 (46.4%)	26.8 ± 12.3		16.1 ± 13.2	

Mean number of nodules per procedure	<3	9 (32.1%)	76.2 ± 14.8	0.017	77.8 ± 13.9	0.021
	≥3	19 (67.9%)	22.2 ± 11.7		21.4 ± 11.4	

Total number of nodules per patient	<3	9 (32.1%)	76.2 ± 14.8	0.006	77.8 ± 13.9	0.023
	≥3 and ≤10	9 (32.1%)	37.0 ± 18.7		41.7 ± 17.3	
	>10	10 (35.7%)	13.6 ± 11.7		0۪ ± 0	

Pulmonary radiotherapy	Present	10 (34.5%)	40.0 ± 15.5	0.346	37.5 ± 16.1	0.334
	Absent	19 (65.5%)	43.7 ± 12.2		43.7 ± 12.2	

**Table 4:** Cox regression analysis of various parameters on survival times after PM and from the time of nodule detection.

Dependant variable: Survival duration after pulmonary metastasectomy (month)	Unstandardized coefficients		Standardized coefficients	t	Sig.
	B	Std. error	Beta		
(Constant)	89.412	36.413		2.455	0.023
Age (years)	−3.741	11.086	−0.058	−0.337	0.739
Sex	5.197	9.528	0.090	0.545	0.591
Presence of metastasis at initial diagnosis	15.135	9.189	0.262	1.647	0.115
Chemotherapy response	5.244	11.824	0.074	0.443	0.662
Interval between metastasis diagnosis and surgery	−27.610	12.218	−0.457	−2.260	**0.035**
Total number of metastatic nodules	−13.959	12.211	−0.243	−1.143	0.266
Number of PM procedures	0.792	9.897	0.014	0.080	0.937
Pulmonary radiotherapy	−15.917	10.116	−0.263	−1.573	0.131
**Dependant variable: Survival duration after nodule detection (month)**	Unstandardized coefficients		Standardized coefficients	t	Sig.
	B	Std. error	Beta		
(Constant)	69.161	39.655		1.744	0.096
Age (years)	2.273	12.073	0.036	0.188	0.853
Sex	5.535	10.376	0.098	0.533	0.600
Presence of metastasis at initial diagnosis	17.490	10.007	0.307	1.748	0.096
Chemotherapy response	10.165	12.877	0.145	0.789	0.439
Interval between metastasis diagnosis and surgery	−19.943	13.306	−0.334	−1.499	0.150
Total number of metastatic nodules	−14.038	13.298	−0.247	−1.056	0.304
Number of PM procedures	−3.541	10.778	−0.062	−0.329	0.746
Pulmonary radiotherapy	−16.566	11.016	−0.278	−1.504	0.148

## References

[1] Rusch VW Pulmonary metastasectomy: a moving target Journal of Thoracic Oncology 2010 5 6 Suppl 2 130 131 10.1097/JTO.0b013e3181dca268 20502246

[2] Nichols FC Pulmonary metastasectomy Thoracic Surgery Clinics 2012 22 1 91 99 10.1016/j.thorsurg.2011.08.017 22108693

[3] Zhao P Jiang Q Xue K Liu X Tian B The role of pulmonary metastasectomy in patients suffering pancreatic ductal adenocarcinoma with lung metastases: a systematic review and meta-analysis Frontiers in Surgery 2025 27 12 1535212 10.3389/fsurg.2025.1535212 40084343 PMC11903735

[4] Corkum KS Craig BT Piche N Pio L Fernandez-Pineda I Current surgical approach to pulmonary metastasectomy Pediatr Blood and Cancer 2025 72 Suppl 2 e31468 10.1002/pbc.31468 39654090

[5] Boam T Rogoyski BG Jawaid W Losty PD Do children with osteosarcoma benefit from pulmonary metastasectomy?: a systematic review of published studies and “real world” outcomes Annals of Surgery 2024 280 2 235 240 10.1097/SLA.0000000000006239 38375639

[6] Richardson WR Progress in pediatric cancer surgery. Recent advances in the surgical management of neoplasms in infants and children Archives of Surgery (Chicago, Ill :1960) 1961 82 641 655 10.1001/archsurg.1961.01300110003001 13741310

[7] Croteau NJ Heaton TE Pulmonary metastasectomy in pediatric solid tumors Children (Basel, Switzerland) 2019 6 1 6 10.3390/children6010006 30626161 PMC6352020

[8] Türk İ Çetin M Gürçay N Sarı N Özkara Ş Bıçakçıoğlu P Tuberculosis mimicking rhabdomyosarcoma metastasis in a pediatric patient Archives of Current Medical Research 2022 3 2 158 162 10.47482/acmr.2022.62

[9] Cooper JD Perelman M Todd TR Ginsberg RJ Patterson GA Pearson FG Precision cautery excision of pulmonary lesions The Annals of Thoracic Surgery 1986 41 1 51 53 10.1016/s0003-4975(10)64495-5 3942431

[10] Marina N Gebhardt M Teot L Gorlick R Biology and therapeutic advances for pediatric osteosarcoma Oncologist 2004 9 4 422 441 10.1634/theoncologist.9-4-422 15266096

[11] Grignani G Palmerini E Dileo P Asaftei SD D’Ambrosio L A phase II trial of sorafenib in relapsed and unresectable high-grade osteosarcoma after failure of standard multimodal therapy: an Italian Sarcoma Group study Annals of Oncology 2012 23 2 508 516 10.1093/annonc/mdr151 21527590

[12] Grier HE Krailo MD Tarbell NJ Link MP Fryer CJ Addition of ifosfamide and etoposide to standard chemotherapy for Ewing’s sarcoma and primitive neuroectodermal tumor of bone The New England Journal of Medicine 2003 348 8 694 701 10.1056/NEJMoa020890 12594313

[13] Raciborska A Bilska K Drabko K Chaber R Pogorzala M Vincristine, irinotecan, and temozolomide in patients with relapsed and refractory Ewing sarcoma Pediatric Blood and Cancer 2013 60 10 1621 1625 10.1002/pbc.24621 23776128

[14] Raney RB Anderson JR Barr FG Donaldson SS Pappo AS Rhabdomyosarcoma and undifferentiated sarcoma in the first two decades of life: a selective review of intergroup rhabdomyosarcoma study group experience and rationale for Intergroup Rhabdomyosarcoma Study V Journal of Pediatric Hematology/Oncology 2001 23 4 215 220 10.1097/00043426-200105000-00008 11846299

[15] Malempati S Hawkins DS Rhabdomyosarcoma: review of the Children’s Oncology Group (COG) Soft-Tissue Sarcoma Committee experience and rationale for current COG studies Pediatric Blood and Cancer 2012 59 1 5 10 10.1002/pbc.24118 22378628 PMC4008325

[16] Tanaka Y Maniwa Y Nishio W Yoshimura M Okita Y The optimal timing to resect pulmonary metastasis European Journal of Cardio-thoracic Surgery 2008 33 6 1135 1138 10.1016/j.ejcts.2008.03.002 18396056

[17] Yamada K Ozawa D Onozato R Suzuki M Fujita A Ojima H Optimal timing for the resection of pulmonary metastases in patients with colorectal cancer Medicine (Baltimore) 2020 99 9 e19144 10.1097/MD.0000000000019144 32118717 PMC7478587

[18] Ichinose J Hashimoto K Matsuura Y Nakao M Akiyoshi T Optimal timing for lung metastasectomy in patients with colorectal cancer Interactive Cardiovascular and Thoracic Surgery 2022 35 4 ivac224 10.1093/icvts/ivac224 35993901 PMC9462424

[19] Detterbeck FC Grodzki T Gleeson F Robert JH Imaging requirements in the practice of pulmonary metastasectomy Journal of Thoracic Oncology 2010 5 6 Suppl 2 134 139 10.1097/JTO.0b013e3181dcf64d 20502248

[20] Krüger M Schmitto JD Wiegmann B Rajab TK Haverich A Optimal timing of pulmonary metastasectomy--is a delayed operation beneficial or counterproductive? European Journal of Surgical Oncology 2014 40 9 1049 1055 10.1016/j.ejso.2014.03.017 24746934

[21] Hamaji M Chen F Miyamoto E Kondo T Ohata K Surgical and non-surgical management of repeat pulmonary metastasis from sarcoma following first pulmonary metastasectomy Surgery Today 2016 46 11 1296 1300 10.1007/s00595-016-1312-x 26892332

[22] Toussi MS Bagheri R Dayani M Anvari K Sheibani S Pulmonary metastasectomy and repeat metastasectomy for soft-tissue sarcoma Asian Cardiovascular and Thoracic Annals 2013 21 4 437 442 10.1177/0218492312462710 24570526

[23] Park JS Kim HK Choi YS Kim K Shim YM et. al Outcomes after repeated resection for recurrent pulmonary metastases from colorectal cancer Annals of Oncology 2010 21 6 1285 1289 10.1093/annonc/mdp475 19861579

[24] Temeck BK Wexler LH Steinberg SM McClure LL Horowitz MA Pass HI Reoperative pulmonary metastasectomy for sarcomatous pediatric histologies The Annals of Thoracic Surgery 1998 66 3 908 913 10.1016/s0003-4975(98)00666-3 9768950

[25] Stephens EH Blackmon SH Correa AM Roth JA Rice DC Progression after chemotherapy is a novel predictor of poor outcomes after pulmonary metastasectomy in sarcoma patients Journal of the American College of Surgeons 2011 212 5 821 826 10.1016/j.jamcollsurg.2011.01.007 21435923

[26] Spratt JS Spratt TL Rates of growth of pulmonary metastases and host survival Annals of Surgery 1964 159 2 161 171 10.1097/00000658-196402000-00001 14119181 PMC1408505

[27] Ahmed G Zamzam M Kamel A Ahmed S Salama A Effect of timing of pulmonary metastasis occurrence on the outcome of metastasectomy in osteosarcoma patients Journal of Pediatric Surgery 2019 54 4 775 779 10.1016/j.jpedsurg.2018.06.019 30005831

[28] Huang YM Hou CH Hou SM Yang RS The metastasectomy and timing of pulmonary metastases on the outcome of osteosarcoma patients Clinical Medicine: Oncology 2009 3 99 105 10.4137/cmo.s531 PMC2872604 20689616

[29] García-Yuste M Cassivi S Paleru C The number of pulmonary metastases: influence on practice and outcome Journal of Thoracic Oncology 2010 5 6 Suppl 2 161 163 10.1097/JTO.0b013e3181dcf787 20502253

[30] Cho JH Kim S Namgung M Choi YS Kim HK The prognostic importance of the number of metastases in pulmonary metastasectomy of colorectal cancer World Journal of Surgical Oncology 2015 13 222 10.1186/s12957-015-0621-7 26205014 PMC4522996

[31] Stork T Boemans R Hardes J Streitbürger A Dirksen U Number of metastases and their response to chemotherapy impact survival of patients with isolated lung metastases from bone-derived sarcoma BMC Cancer 2021 21 1 375 10.1186/s12885-021-08073-3 33827467 PMC8028220

[32] Smolle MA Kogler A Andreou D Scheipl S Bergovec M Prognostic impact of pulmonary metastasectomy in bone sarcoma patients: a retrospective, single-centre study Cancers (Basel) 2023 15 6 1733 10.3390/cancers15061733 36980620 PMC10046382

[33] Lin AY Kotova S Yanagawa J Elbuluk O Wang G Risk stratification of patients undergoing pulmonary metastasectomy for soft tissue and bone sarcomas The Journal of Thoracic and Cardiovascular Surgery 2015 149 1 85 92 10.1016/j.jtcvs.2014.09.039 25312228

[34] Liu Z Yin J Zhou Q Yang J Zeng B Survival after pulmonary metastasectomy for relapsed osteosarcoma The Journal of Thoracic and Cardiovascular Surgery 2022 163 2 469 479 10.1016/j.jtcvs.2020.10.137 33349447

[35] Karpelowksy J Seitz G A surgical approach to pulmonary metastasis in children Surgical Oncology Clinics of North America 2021 30 2 389 399 10.1016/j.soc.2020.11.007 33706907

[36] da Nobrega Oliveira REN de Andrade Pontual Peres C Oliveira AC Onyeji P Kemczenski F Comparative outcomes of video-assisted thoracic surgery versus open thoracic surgery in pediatric pulmonary metastasectomy: a systematic review and meta-analysis Pediatric Surgery International 2024 41 1 34 10.1007/s00383-024-05934-3 39699640

[37] Traynor MD Brar GD Bruno FP Iyer G Ishitani MB Pulmonary metastasectomy in pediatric patients: a comparison of open and thoracoscopic approaches Journal of Laparoendoscopic and Advanced Surgical Techniques 2022 32 1 64 71 10.1089/lap.2021.0439 34783259

[38] Lautz TB Krailo MD Han R Heaton TE Dasgupta R Doski J Current surgical management of children with osteosarcoma and pulmonary metastatic disease: a survey of the American Pediatric Surgical Association Journal of Pediatric Surgery 2021 56 2 282 285 10.1016/j.jpedsurg.2020.09.060 33558032

[39] Lautz TB Farooqui Z Jenkins T Heaton TE Doski JJ Thoracoscopy vs thoracotomy for the management of metastatic osteosarcoma: a pediatric surgical oncology research collaborative study International Journal of Cancer 2021 148 5 1164 1171 10.1002/ijc.33264 32818304

[40] Yenigün BM Yüksel C Kahya Y Görgüner F Çoruh Gürsoy A Effectiveness of intraoperative bimanual palpation in metastatic tumors of lung Turk Gogus Kalp Damar Cerrahisi Dergisi 2020 28 4 662 668 10.5606/tgkdc.dergisi.2020.20429 33403140 PMC7759051

